# Utilizing Carbonated Reclaimed Water as Concrete Mixing Water: Improved CO_2_ Uptake and Compressive Strength

**DOI:** 10.3390/ma19010076

**Published:** 2025-12-24

**Authors:** Hoon Moon, Muhammad Haseeb Zaheer, Indong Jang, Gi-Joon Park, Jung-Jun Park, Sehee Hong, Namkon Lee

**Affiliations:** 1 Department of Structural Engineering, Korea Institute of Civil Engineering and Building Technology, 283 Goyangdae-ro, Ilsanseo-gu, Goyang-si 10223, Gyeonggi-do, Republic of Korea; hmoon@kict.re.kr (H.M.); joon7767@kict.re.kr (G.-J.P.); shhong@kict.re.kr (S.H.); 2 Department of Civil and Environmental Engineering, University of Science and Technology, 217 Gajeong-ro, Yuseong District, Daejeon 34113, Republic of Korea

**Keywords:** reclaimed water, carbonation, CO_2_ uptake, mixing water, compressive strength

## Abstract

This study investigates the carbonation degree of reclaimed water (RW) and its potential use as mixing water for cementitious materials under controlled laboratory conditions using a simplified CO_2_ injection method. To reproduce the chemical environment of actual RW, a synthetic reclaimed water (SRW) system with a cement-to-sand ratio of 8:2 was prepared and used throughout the evaluation. Thermogravimetric analysis revealed that the cementitious solids suspended in SRW exhibit high reactivity with CO_2_, achieving a net CO_2_ uptake of 16.8%, equivalent to 8.31 g of CO_2_ sequestered per kilogram of RW. The use of untreated RW as mixing water slightly reduced flowability and increased superplasticizer demand compared with distilled water, whereas carbonation treatment of RW improved workability and mitigated the rapid initial setting typically observed with untreated RW. Notably, replacing 3% of the cement with carbonated RW solids did not cause any reduction in compressive strength, indicating that the carbonated solids can be incorporated without compromising mechanical performance. These results confirm that the CaCO_3_ formed during RW carbonation remains stably retained within mortar and concrete, demonstrating the feasibility of using carbonated RW as a dual-function material—serving both as mixing water and as a medium for CO_2_ sequestration.

## 1. Introduction

Global efforts to mitigate climate change and achieve carbon neutrality are accelerating, creating a pressing need for the development of carbon-reduction technologies across industrial sectors. Among these, the construction industry is recognized as a major emitter of greenhouse gases, with cement production alone accounting for approximately 7–8% of global CO_2_ emissions [1,2].

Cement manufacturing requires a high-temperature calcination process at around 1500 °C, during which a considerable volume of CO_2_ is released. This process is widely acknowledged as one of the key contributors to the rising levels of greenhouse gases and, consequently, to global climate change [3,4]. Given its indispensable role as a binding material in construction, cement remains essential to infrastructure development. Therefore, the construction industry urgently needs to adopt carbon-reduction strategies that not only reduce CO_2_ emissions but also facilitate the effective utilization of emitted CO_2_.

During the production of ready-mixed concrete, large volumes of wash water are generated, commonly referred to as reclaimed water (RW). Reclaimed water primarily arises from the washing of batch plant mixers and the cleaning of the interior drums of returned agitator trucks after concrete placement. The reclaimed water undergoes a separation process to remove coarse and fine aggregates sequentially, after which it is stored in designated tanks.

Reclaimed water consists mainly of water, cement, and fine particles, and is characterized by a strongly alkaline pH ranging from 12 to 13 [5]. Due to its high pH and substantial solid content of 5 to 30%, the uncontrolled discharge of reclaimed water can pose significant environmental risks, particularly in terms of water and soil contamination [6]. To mitigate such concerns, most ready-mixed concrete plants store reclaimed water in tanks and reuse it as mixing water for concrete production. However, direct reuse without appropriate treatment may adversely affect the fresh and hardened properties of concrete. In addition, the high alkalinity and solid content of reclaimed water have been shown to negatively influence workability, setting time, and compressive strength [5,7].

To address these concerns, several international standards specify limits on the allowable solid content in reclaimed water when used as mixing water. ASTM C1602 stipulates a maximum total solids content of 50,000 ppm (50 g/L) [8], while EN 1008 limits solids to less than 1% of the aggregate mass [9]. In Japan, JIS A 5308, and in South Korea, KS F 4004, restrict the solids content to less than 3% of the total binder mass when reclaimed water is used in concrete mixing [10,11]. These quality standards serve as baseline guidelines to prevent deterioration in concrete performance when reclaimed water is used in mix design.

In this study, a carbonation treatment of reclaimed water was applied to resolve issues of performance degradation or variability in concrete associated with its use, and to explore the feasibility of utilizing reclaimed water as a CO_2_ sink capable of storing a significant amount of CO_2_ within concrete. To evaluate its carbon uptake potential, actual reclaimed water (RW) collected from ready-mixed concrete production was carbonated using CO_2_ gas under controlled conditions. The CO_2_-treated reclaimed water was reused as mixing water to investigate the subsequent effects on concrete properties. The characteristics of RW under various CO_2_ injection conditions are first analyzed, followed by an assessment of fresh and hardened properties—including workability, setting time, and compressive strength—when the treated RW is incorporated into concrete mixtures. This approach enables evaluation of whether CO_2_ treatment of RW can contribute to performance enhancement in concrete. Furthermore, by analyzing the long-term potential for the storage of calcium carbonate precipitated during the CO_2_ reaction process, this study explores the feasibility of the proposed method as a carbon mitigation strategy within the construction sector, thereby contributing to the broader goal of achieving carbon neutrality.

## 2. Experimental Procedure

### 2.1. Materials

The cement used in this study was Type I ordinary Portland cement manufactured by Sungshin Cement Co., Ltd. The ISO standard sand (in accordance with ISO 679) was used as the fine aggregate for mortar mixtures to minimize external variability. For concrete mixtures, both fine and coarse aggregates were sourced locally from regional suppliers. The chemical and mineralogical compositions of ordinary Portland cement are listed in Table 1.

### 2.2. CO_2_-Enriched Nano Bubble Water

Nanobubbles with diameters smaller than 500 nm are known to maintain stability over extended periods due to their high internal pressure and interfacial charge. Owing to these unique physicochemical properties, nanobubble technology has gained increasing attention for potential applications in various industrial and environmental fields [12]. In particular, nanobubbles can enhance gas solubility in water while simultaneously encapsulating gases in microbubble form, enabling their long-term retention in aqueous systems [13,14].

Based on these advantages, recent studies have explored the use of CO_2_-enriched nanobubble water in cementitious systems, either as mixing or curing water, to promote in situ CO_2_ sequestration within the cement matrix [15,16,17]. These studies demonstrated that CO_2_ nanobubbles can contribute not only to mechanical performance improvement but also to enhanced carbon capture and storage in concrete.

In this work, nanobubble water was employed solely as a laboratory-scale medium to enable controlled dissolution of CO_2_ for fundamental reactivity analysis; in contrast, the carbonation of reclaimed water was conducted by direct CO_2_ gas injection to reflect practical application conditions.

The carbonation degree between fresh ordinary Portland cement powder and dissolved CO_2_ in the nanobubble water was evaluated by preparing a high-concentration CO_2_ nanobubble solution and reacting it with cement particles. The CO_2_ nanobubble solution was produced by injecting CO_2_ gas at a flow rate of 5 L/min into tap water of 100 L using a nanobubble generator until the CO_2_ concentration in the solution reached 1400 mg/L. The CO_2_ concentration of the nanobubble solution was measured using a CO_2_ concentration meter (CGP-31, TOADKK, Japan) based on a diaphragm-type glass electrode. The resulting CO_2_ concentration was measured at 1351 mg/L. After 24 h of sealed storage, the CO_2_ concentration remained at 1340 mg/L, confirming the stability of CO_2_ within the nanobubble solution.

Nanobubbles have been reported to remain stable for up to six months, and over 50% of the initially captured CO_2_ can still be retained after two weeks [18]. These properties make nanobubble water particularly suitable for quantifying CO_2_ uptake resulting from reactions with cementitious solids in aqueous environments, especially in complex systems such as reclaimed water, which contains suspended fine cement particles and hydration products.

**Figure 1:**
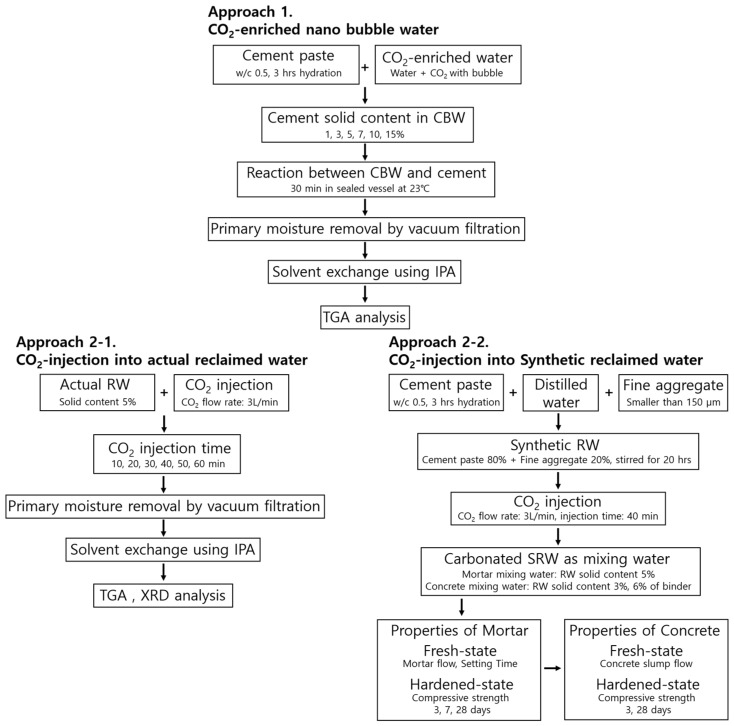
Research approach diagram.

To quantitatively assess the carbonation degree between fresh ordinary Portland cement powder and CO_2_ nanobubble water, a series of cement suspensions with varying solid contents (1, 3, 5, 7, 10, and 15 wt.%, based on cement powder) were prepared and reacted with CO_2_-enriched nanobubble water (Figure 1). For comparison, a control sample composed of 5 wt.% cement mixed with distilled water was analyzed under the same conditions.

### 2.3. Actual and Synthetic Reclaimed Water

Reclaimed water (RW), generated during the production of ready-mixed concrete, exhibits considerable variation in its chemical and physical composition depending on factors such as point of origin, time of collection, and the types of raw materials used. These compositional fluctuations can influence not only the carbonation degree of the RW but also the resulting properties of concrete when it is used as mixing water.

Preliminary tests (Approach 1) were conducted to assess the carbonation degree and compositional variability of RW. Three types of actual RW samples (RW-A, RW-B, and RW-C) were collected from different ready-mixed concrete batch plants. Each sample underwent carbonation under identical temperature conditions of 20 °C by injecting CO_2_ gas, and the carbonation degree of the solids was quantitatively evaluated using thermogravimetric analysis (Figure 1). This approach allowed for a comparison of the carbonation behavior among the actual RW samples with different compositions, and provided baseline values for subsequent experiments.

Based on the preliminary experiments, synthetic reclaimed water was prepared in the laboratory to analyze the carbonation degree of RW and assess the feasibility of its applicability as mixing water in concrete. To obtain a synthetic RW with a composition and reactivity comparable to those of actual reclaimed water, the ratio of cement to fine aggregate was systematically varied and its carbonation behavior was evaluated. Through this comparative analysis, a cement-to-sand ratio of 2:8 was identified as providing the closest correspondence to the carbonation reactivity and solid-phase characteristics observed in actual RW. Accordingly, the synthetic RW was formulated by mixing cement with fine aggregate particles smaller than 150 μm at a fixed ratio of 2:8, designed to replicate the solid components and particle-size distribution observed in the actual RW.

All samples (Approach 2) were first hydrated at a water-to-cement ratio (*w*/*c*) of 0.5 for 3 h, followed by an extended mixing period of 20 h to promote further hydration (Figure 1). After this pre-treatment, gaseous CO_2_ was injected to induce the carbonation reaction. An additional 20 h stirring step was applied after the initial 3 h hydration to adjust the Ca(OH)_2_ content to levels comparable to those in actual reclaimed wash water. In the TG analysis of the sample hydrated for only 3 h, no mass loss corresponding to the thermal decomposition of Ca(OH)_2_ was observed in the 400–500 °C range. To better replicate the chemical state of actual reclaimed water, the hydration process was extended to 20 h to ensure sufficient formation of Ca(OH)_2_, thereby enabling more realistic and representative carbonation behavior during subsequent CO_2_ exposure.

Actual RW samples and synthetic RW samples were tested under the same protocols, and their carbonation degree and physical properties was comparatively evaluated.

### 2.4. Mix Proportions of Mortar and Concrete

A water-to-cement (*w*/*c*) ratio of 0.5 study included different types of water, mortar specimens, and concrete specimens prepared under various mixing conditions. To clearly define the experimental groups and ensure consistent labeling throughout the manuscript, all investigated samples and their corresponding short names are summarized in Table 2. This table provides an overview of the water types used as mixing water as well as the mortar and concrete mixtures prepared with synthetic and carbonated reclaimed water.

Table 3 and Table 4 present the mixture proportions for the mortar and concrete specimens. To evaluate the influence of reclaimed water (RW) and CO_2_-treated reclaimed water as mixing water, a series of mortar and concrete samples were prepared under various mixing conditions using the sample labels listed in Table 2.

A water-to-cement ratio (*w*/*c*) of 0.5 was adopted as the baseline condition for both mortar and concrete mixtures. This value is one of the most commonly used water–cement ratios in cement-based material research, as it provides a balanced condition between workability and hydration. In particular, for mortar mixtures, a *w*/*c* ratio of 0.5 is specified in ISO 679 for the preparation of standard cement mortar specimens, ensuring reproducibility and facilitating comparison with previous studies. Applying the same *w*/*c* ratio to concrete mixtures allowed the effects of mixing water type to be evaluated under consistent reference conditions.

The solid content of reclaimed water was set at 3 wt.% of the binder mass, in accordance with the limits prescribed by JIS A 5308 (Japan) and KS F 4004 (South Korea). Although this value is slightly higher than the limit recommended by ASTM C1602, the deviation is not significant enough to cause noticeable changes in the physical properties of concrete.

For mortar mixtures, all sample labels are prefixed with “M-”. M-DW denotes mortar specimens prepared with distilled water as the control. M-SRW refers to mortar specimens prepared with synthetic reclaimed water containing 3 wt.% solids relative to the binder mass, while M-cSRW refers to specimens prepared with carbonated synthetic reclaimed water containing the same solid content.

For concrete mixtures, all sample labels are prefixed with “C-”. C-DW denotes the control concrete prepared with distilled water. C-SRW3% and C-cSRW3% represent concrete mixtures prepared with synthetic reclaimed water and carbonated synthetic reclaimed water containing 3 wt.% solids, respectively. In addition, C-cSRW3%-R denotes the concrete mixture in which the solid fraction contained in the carbonated synthetic reclaimed water was considered as a partial replacement of cement, corresponding to a 3% cement replacement. For the C-cSRW3%-R mixture, the cement content was reduced by an amount equivalent to the solid content contained in the reclaimed water (11.1 kg/m^3^). This mixture was designed to evaluate whether concrete incorporating reclaimed water could achieve compressive strength comparable to that of the control mixture (C-DW) despite a reduced cement dosage. Maintaining comparable strength under this condition would indicate potential economic benefits as well as a reduction in cement-related carbon emissions.

Furthermore, concrete mixtures containing 6 wt.% solid content, namely C-SRW6% and C-cSRW6%, were designed to examine the effects of reclaimed water solid contents exceeding the limits specified in relevant standards on the fresh and hardened properties of concrete.

The aggregate proportions for mortar and concrete were designed differently to reflect their distinct material systems and testing objectives. Mortar mixtures followed standard mortar mixture design practices, whereas concrete mixtures were proportioned according to conventional concrete mixture design principles. Accordingly, differences in aggregate ratios between mortar and concrete are inherent to their respective mixture design methodologies and do not affect the comparative evaluation of reclaimed water effects within each material system.

### 2.5. X-Ray Diffraction (XRD) Analysis

The mineralogical compositions of the actual reclaimed water and the synthetic reclaimed water were analyzed by X-ray diffraction (XRD). The XRD measurements were carried out using a benchtop X-ray diffractometer (Aeris, Malvern Panalytical).

### 2.6. Thermogravimetric Analysis

Thermogravimetric analysis (TGA) was performed using a thermogravimetric analyzer (TGA N-1000, Sinco M&T) to evaluate the thermal decomposition behavior of the samples. Thermogravimetric analysis (TGA) was conducted to quantify the amount of CaCO_3_ formed through the reaction between CO_2_ and the cementitious solids present in reclaimed water. Calcium carbonate undergoes thermal decomposition within the temperature range of approximately 500–850 °C, following the reaction CaCO_3_ → CaO + CO_2_. The release of CO_2_ gas results in a measurable mass loss of the sample. Therefore, the mass loss observed in this temperature range corresponds to the decomposition of CaCO_3_ formed during the aqueous carbonation reaction, serving as a quantitative indicator of CO_2_ fixation between the solid content and the injected CO_2_.

The TG analysis was performed according to ASTM C1910, using a heating rate of 10 °C/min up to 950 °C. The CO_2_ uptake was calculated based on the mass loss specifically occurring between 500 °C and 850 °C.

Cement-based solids in reclaimed water may contain a small amount of CaCO_3_ formed through natural carbonation with atmospheric CO_2_ during production or storage. Accordingly, the net amount of CO_2_ uptake was calculated by subtracting the CO_2_ release of the reference sample from that of the test sample, as shown in Equation (1):

ΔCO_2_ = CO_2,sample_ − CO_2,reference_ (1)

CO_2,sample_ = Measured CO_2_ release (%) from the RW sample reacted with CO_2_
CO_2,reference_= Measured CO_2_ release (%) from the reference sample containing only cement and distilled water
ΔCO_2_ = Net amount of CO_2_ fixed through the reaction between RW solids and CO_2_ (%)

### 2.7. Physical Properties of Mortar and Concrete

Mortar specimens were cast in 50 mm × 50 mm × 50 mm cube molds. Flow and initial setting time were measured to investigate the effect of RW on the workability and setting characteristics of mortar. The flowability of mortar was evaluated in accordance with ASTM C1437, which specifies a standard flow table test for assessing the consistency of hydraulic cement mortars based on their spread diameter after a prescribed number of drops. The setting time was measured using a Vicat needle in accordance with ASTM C191, which determines the initial and final setting times of cementitious materials based on penetration resistance. An automated Vicat apparatus (VICAMATIC-3, Controls Group, Italy) was used to ensure consistent testing conditions.

Compressive strength tests of mortar were conducted at curing ages of 3, 7, and 28 days in accordance with ASTM C109, which provides a standardized procedure for determining the compressive strength of cement mortars using prismatic specimens. For each mixture and test age, six specimens were tested, and the average value was reported.

Concrete specimens were cast in cylindrical molds with dimensions of 100 mm in diameter and 200 mm in height for compressive strength testing. The slump test was conducted to evaluate the workability and flowability of concrete incorporating carbonated reclaimed water. Compressive strength tests of concrete were performed in accordance with ASTM C39, which specifies the procedure for measuring the compressive strength of cylindrical concrete specimens under uniaxial loading. For each mixture and test age, six cylindrical specimens were tested, and the average compressive strength was used for analysis.

## 3. Results and Discussion

### 3.1. Aqueous CO_2_ Reactivity of Cement

**Figure 2:**
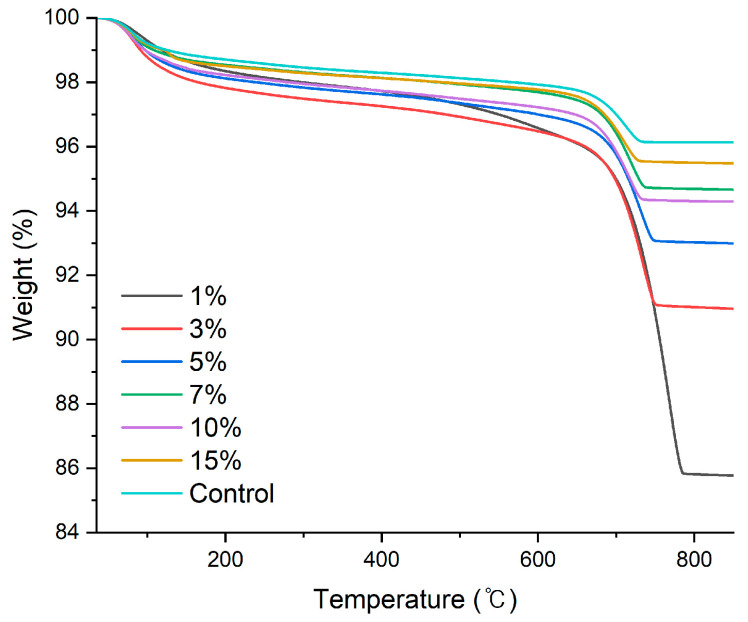
TG analysis of cement powders reacted with CO_2_-enriched nano-bubble water at different cement solids contents (1, 3, 5, 7, 10, and 15 wt.%), together with a control sample.

This experiment was designed as a model system to investigate the fundamental aqueous carbonation reactivity between cement powder and dissolved CO_2_ under controlled conditions. Figure 2 presents the TGA results of the cement powders reacted with CO_2_-enriched nanobubble water (CBW). A distinct mass loss was observed in all specimens within the temperature range of approximately 500–850 °C, which is attributed to the thermal decomposition of CaCO_3_. Notably, samples with lower cement contents (1–3 wt.%) exhibited a greater percentage of mass loss, suggesting higher degree of carbonation due to the abundance of dissolved CO_2_ relative to the available Ca^2+^. On the other hand, as the cement content increased, the relative mass loss gradually decreased, suggesting that under a constant CO_2_ concentration, an increase in reactive Ca^2+^ leads to lower carbonation efficiency.

Table 5 summarizes the CO_2_ uptake results calculated from thermogravimetric (TG) analysis. After correcting for the mass loss observed in the control sample, the net CO_2_ uptake—attributed solely to the reaction with dissolved CO_2_—was found to be highest (9.52%) at cement solid content of 1 wt.%. As the cement content increased, the CO_2_ uptake exhibited a decreasing trend. This result indicates that under a constant CO_2_ concentration, an increase in the amount of cement leads to a lack of available CO_2_ per unit of Ca^2+^, thereby reducing the overall reaction efficiency. Thus, the carbonation degree in the CBW appears to be governed by the stoichiometric ratio between dissolved CO_2_ and available Ca^2+^ ions rather than by the absolute amount of cement.

The net CO_2_ uptake was normalized by the volume of solution and the values ranged from 705 to 1180 mg/L, as listed in Table 5, showing relatively small variation. This indicates that the carbonation efficiency per unit volume of solution remains fairly constant. In the CBW, the carbonation degree was primarily governed by the amount of dissolved CO_2_, rather than the cement content, due to the limited availability of CO_2_.

The pH of the distilled water was initially measured at 7.31, while the CBW showed a pH of 5.07, reflecting the acidic characteristics of dissolved CO_2_. The value after the reaction between the cement solids and CBW ranged from 10.75 to 11.82. The increase in pH is attributed to the release of OH^−^ ions during the hydration of cement, and the simultaneous removal of free CO_2_ via its reaction with Ca^2+^ ions, which neutralizes the acidic nature of the initial solution.

As the cement solids content increases, the amount of calcium hydroxide (Ca(OH)_2_) available to react with CO_2_ also increases. However, since the total amount of dissolved CO_2_ in the CBW remains constant, the limited absolute amount of CO_2_ relative to the available calcium ions restricts the extent of the carbonation reaction. As a result, unreacted Ca(OH)_2_ remains in the solution, maintaining a high level of alkalinity and resulting in a relatively elevated pH. In contrast, when the solids content is low, the available CO_2_ is sufficient relative to the cement content, resulting in more effective consumption of Ca(OH)_2_. This leads to a lower OH^−^ concentration and, consequently, a slightly reduced pH. Thus, it suggests that the final pH and the degree of alkalinity retention in the solution are governed by the relative reaction ratio between the cement solids and the dissolved CO_2_. The CO_2_-to-solids ratio plays a critical role in determining both the extent of the carbonation reaction and the system’s ability to recover alkalinity.

The results in Section 3.1 clearly demonstrated the reactivity between the dissolved CO_2_ in the CO_2_ nanobubble solution and cement. Notably, effective carbonation reactions were achieved under highly dilute conditions—where the water-to-cement ratio was high—provided that a sufficient concentration of dissolved CO_2_ was available to react with Ca^2+^ ions.

Based on these results, synthetic reclaimed water with controlled composition was prepared to analyze its reactivity with CO_2_ prior to evaluating the carbonation degree of actual wash water generated during concrete production. The objective of this experiment was to quantitatively investigate the formation of calcium carbonate via reactions between dissolved CO_2_ and fine cement particles suspended in actual RW-like conditions.

### 3.2. Analysis of CO_2_ Reactivity of Actual and Synthetic Reclaimed Water

Based on the findings in Section 3.1, synthetic reclaimed water with controlled composition was prepared to further investigate CO_2_ reactivity under conditions representative of actual wash water.

To focus specifically on the CO_2_ reactivity of the reclaimed water, the synthetic reclaimed water excluded chemical admixtures and supplementary cementitious materials, and cement fines and fine aggregate dust were used as part of solid components. The fine aggregate dust was included to replicate the physical characteristics of actual reclaimed water. The synthetic RW was prepared with five different weight ratios of cement to sand fines: 10:0, 9:1, 8:2, 7:3, and 6:4. These compositional variations allowed for a systematic analysis of carbonation degree under different particle size distributions.

Although the carbonation degree of cement fines reacted with CO_2_ nanobubble solution was evaluated in Section 3.1, the amount of dissolved CO_2_ available in the nanobubble water was inherently limited. As a result, the dissolved CO_2_ alone was insufficient to induce complete carbonation of the solid phase under the tested conditions. Accordingly, to increase the extent of the reaction between CO_2_ and suspended solids in reclaimed water, the CO_2_ supply method was modified in Section 3.2 from nanobubble-based dissolution to continuous gas-phase CO_2_ injection. This modification ensured a sustained availability of CO_2_ throughout the reaction process, thereby overcoming the limitation associated with finite dissolved CO_2_ and enabling more extensive CaCO_3_ formation.

#### 3.2.1. Carbonation Degree of Reclaimed Water

To evaluate the intrinsic CO_2_ reactivity of synthetic and actual RW, a carbonation experiment was conducted using the actual RW (RW-C sample), which contained approximately 11% solids without any prior adjustment of solids content. A volume of 1000 mL of RW-C was placed in the beaker and continuously stirred at 450 rpm using a magnetic stirrer. Gaseous CO_2_ was injected at a flow rate of 3 L/min, and samples were extracted at 10 min intervals for thermogravimetric analysis (TGA) and pH measurement.

**Figure 3:**
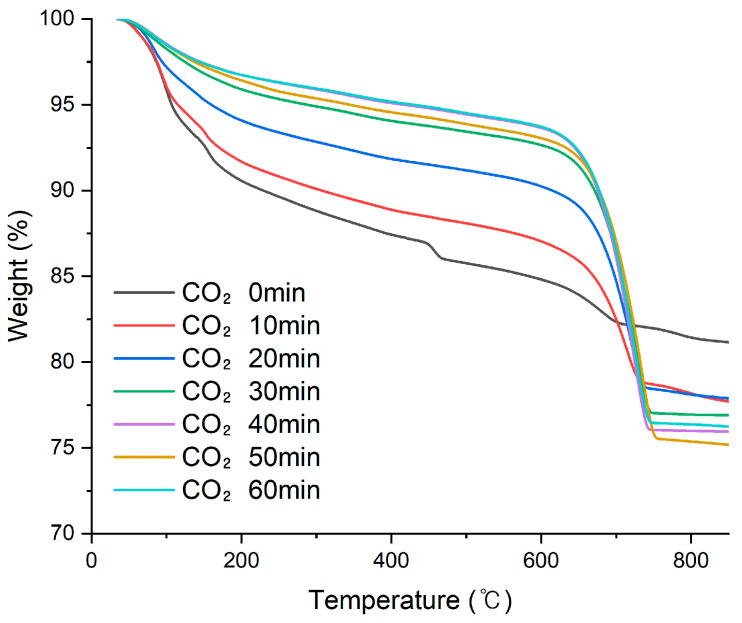
TG analysis of RW-C at different CO_2_ injection time.

As shown in the TGA curves (Figure 3), the sample before CO_2_ injection exhibited a distinct weight loss within the temperature range of 450–550 °C, corresponding to the thermal decomposition of calcium hydroxide (CH). Whereas, no significant weight loss was observed in this temperature range after just 10 min of CO_2_ injection, and this trend continued up to 60 min. These results indicate that CH was rapidly converted into calcium carbonate (CaCO_3_) during the initial phase of CO_2_ exposure, highlighting the high reactivity of RW solids and their ability to undergo carbonation within a short reaction time.

**Figure 4:**
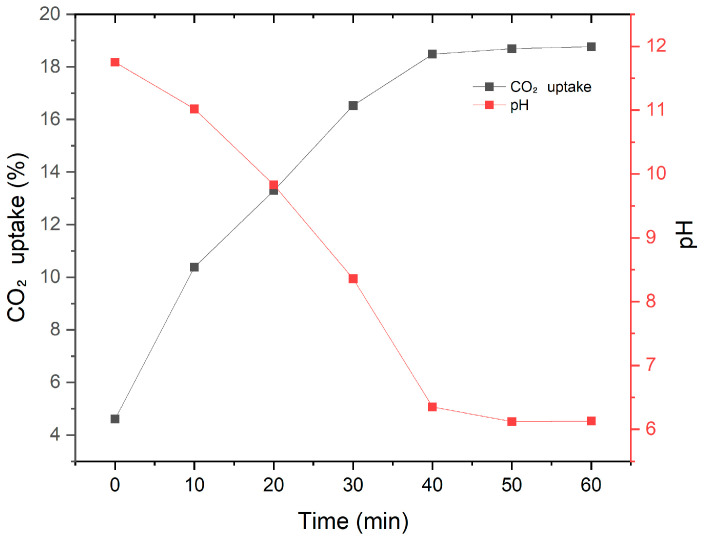
CO_2_ uptake (%) and pH of RW-C as a function of CO_2_ injection time.

As shown in Figure 4, the CO_2_ uptake (%) before CO_2_ injection was 4.61%, which steadily increased to 18.49% after 40 min of CO_2_ injection. The CO_2_ uptake plateaued thereafter, reaching 18.69% at 50 min and 18.67% at 60 min. The pH of the solution decreased gradually from an initial value of 11.75 to 8.36 for 30 min. A sharp drop was observed thereafter, with the pH reaching 6.12 at 40 min and remaining nearly constant afterward. This reduction in pH is attributed to the precipitation of CaCO_3_ resulting from the reaction between dissolved CO_2_ and Ca^2+^ ions in the RW. Moreover, the dissolved CO_2_ underwent equilibrium transformations into carbonic acid (H_2_CO_3_), bicarbonate (HCO_3_^−^), and carbonate ions (CO_3_^2−^), contributing to the acidification of the aqueous phase. As the reaction progressed, the OH^−^ concentration declined and the buffer capacity of the solution weakened, leading to a rapid pH decrease [19].

Notably, a critical transition was observed at 20 min of CO_2_ injection, where the CO_2_ uptake reached 13.29% and the pH dropped to 9.83. From this point onward, the pH declined rapidly while the increase in CO_2_ uptake began to decelerate, indicating the onset of equilibrium-limited behavior. This means that the initial reaction between Ca^2+^ and CO_2_ in the solution was temporarily buffered, but as carbonation progressed, the buffer capacity was exhausted, leading to a stagnation in further reaction.

A similar response was observed in the RW-A sample. The solid content was adjusted to 5% and CO_2_ was injected at a rate of 3 L/min for 30 min, 1 h, and 3 h. The TGA results are summarized in Table 6. The CO2 uptake reached 22.12% at 30 min and showed only marginal increases at 1 h (22.56%) and 3 h (22.92%), confirming that most of the reactive solids carbonated rapidly within 30 min.

The results of both RW-A and RW-C indicate that the carbonation of reclaimed water proceeds rapidly during the early stage. Most of the CH completed its reaction within 10 min, and the carbonation of the solid phase did not progress beyond 40 min. Beyond this point, continued CO_2_ injection contributes little to further carbonation and instead promotes acidification of the aqueous phase, which may lead to dissolution of CaCO3 under the conditions of pH 6.5. Therefore, the optimal CO_2_ injection duration for the carbonation of reclaimed water solids was in the range of 30–40 min to ensure process efficiency.

**Figure 5:**
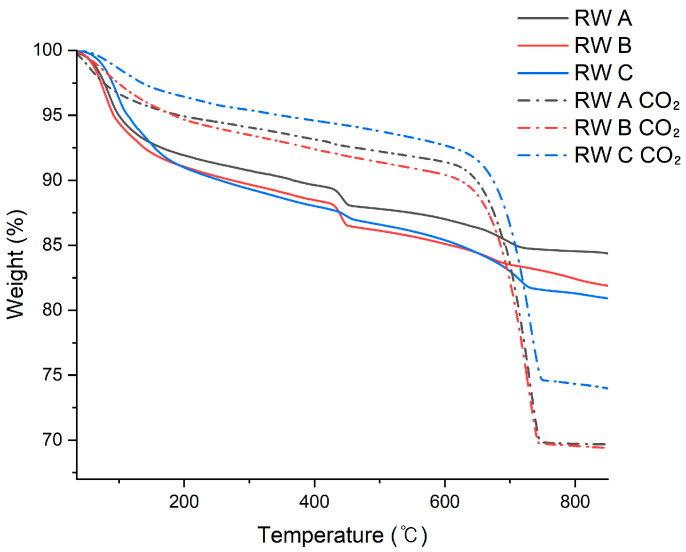
TG analysis of reclaimed water samples before and after carbonation.

Figure 5 presents the TG results of the actual RW samples (RW-A, RW-B, and RW-C) before and after carbonation. To compare CO_2_ reactivity, the solid components were extracted from each actual reclaimed water sample, then mixed with distilled water to adjust the solid content to a fixed value of 5 wt.%. This approach reduced variations in the initial solid concentration and composition of the RW samples, thereby enabling quantitative comparisons.

After the carbonation reaction during 40 min, the mass loss in the temperature range of 500–850 °C was analyzed. As summarized in Table 7, the CO_2_ uptake was 19.13% for RW-A, 17.77% for RW-B, and 14.18% for RW-C. The CO_2_ uptake per 1 L of reclaimed water for RW-A, RW-B, and RW-C was 9.56 g, 8.89 g, and 6.99 g, respectively. This result indicates that the differences in the composition of solid constituents—particularly the type and availability of Ca^2+^ sources—can significantly influence the carbonation efficiency. The average CO_2_ uptake (%) of the three samples was calculated to be approximately 17.01%, which was used as a reference for designing the composition of the synthetic reclaimed water in subsequent experiments.

#### 3.2.2. Carbonation Degree of Synthetic Reclaimed Water

**Figure 6:**
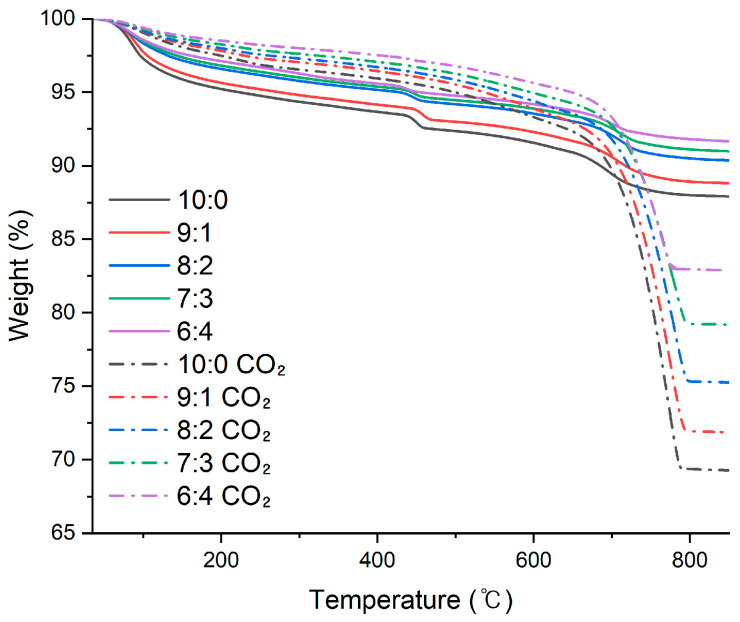
TG analysis before and after carbonation for synthetic reclaimed water samples at different cement-to-sand ratios.

Figure 6 presents the TG curves of synthetic reclaimed water samples before and after carbonation, prepared with a fixed solid content of 5 wt.% and varying cement-to-sand (C:S) ratios. CO_2_ injection induced carbonation reactions, as evidenced by the disappearance of CH-related peaks, confirming that complete carbonation was achieved.

Table 8 summarizes the TG analysis results for the synthetic reclaimed water samples after carbonation. The net CO_2_ uptake exhibited a clear increasing trend with higher cement content. The C:S = 10:0 mixture showed the highest net CO_2_ uptake at 21.28%, whereas the lowest value of 10.80% was observed for the C:S = 6:4 mixture. A similar trend was found in the CO_2_ uptake per 1 L of RW (mg/L), which also decreased as the proportion of cement decreased. On the other hand, net CO_2_ uptake per cement content in 1 L of RW remained relatively consistent for all mixtures, ranging from approximately 17.9% to 21%. These results indicate that although the net CO_2_ uptake decreases with lower cement proportions, the carbonation efficiency per unit cement content in a given volume of RW does not significantly decrease. As long as a sufficient supply of CO2 is maintained, the carbonation reaction can proceed in a stable and effective manner regardless of the presence of inert fines such as sand particles.

In Table 8, the CO_2_ uptake showed a clear proportional relationship with cement content. The CO_2_ uptake (16.77%) and the net CO_2_ uptake per 1 L of RW (8305 mg/L) for the C:S = 8:2 sample were most similar to the average values (17.04% and 8505 mg/L, respectively; Table 7) of actual reclaimed water. Accordingly, the C:S = 8:2 condition was selected as the representative composition for the synthetic reclaimed water and was subsequently used as the mixing water for producing mortar and concrete specimens.

**Figure 7:**
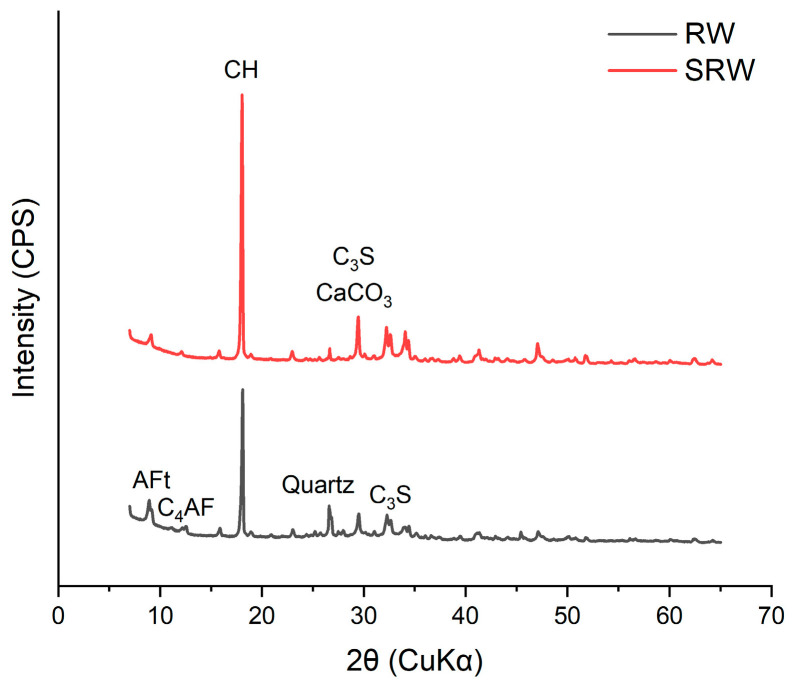
XRD analysis of solids from synthetic reclaimed water (SRW) and actual reclaimed water (RW).

To further verify whether this representative synthetic reclaimed water also reproduces the mineralogical characteristics of actual reclaimed water, XRD analysis was conducted on the solid fractions extracted by filtration. The XRD patterns of solids obtained from both synthetic and actual reclaimed water are presented in Figure 7. In both cases, pronounced diffraction peaks corresponding to calcium hydroxide (CH) were observed, along with distinguishable reflections associated with AFt phases. These results indicate that the synthetic reclaimed water with a C:S ratio of 8:2 not only exhibits carbonation behavior comparable to that of actual reclaimed water but also closely replicates its mineralogical characteristics.

### 3.3. Physical Properties of Mortar Using Synthetic Reclaimed Water

**Figure 8:**
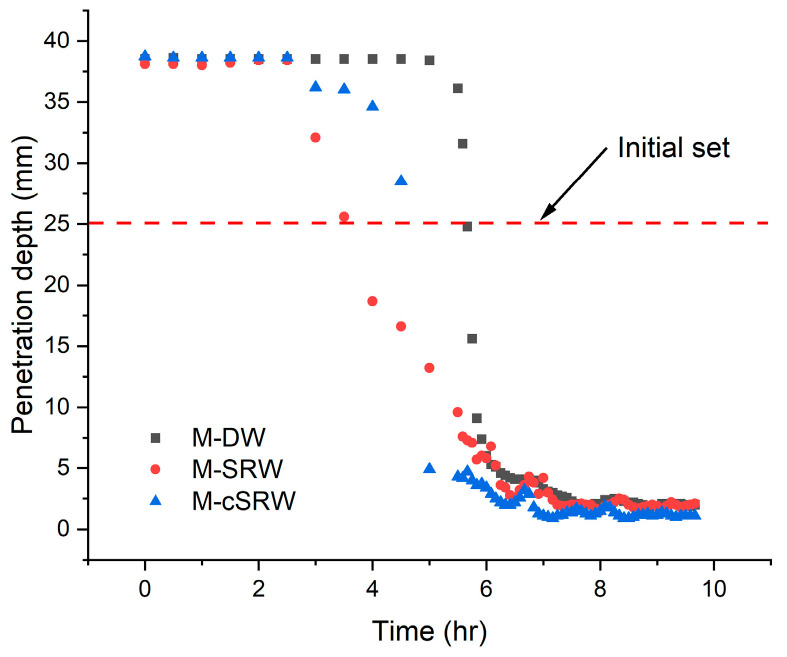
Initial setting of mortar using the Vicat apparatus.

Figure 8 presents the initial setting time of M-DW, M-SRW, and M-cSRW. Table 9 summarizes the corresponding flow values and initial setting times. The initial setting time was defined as the point at which the Vicat needle penetration reached 25 mm, calculated using linear interpolation.

As shown in Table 9, M-SRW exhibited a reduced flow of 180.7 mm compared to 208.6 mm for M-DW. In Figure 8, the initial setting time of M-SRW was markedly shortened to 3.09 h, compared to 5.66 h for M-DW. This acceleration in setting behavior is likely associated with enhanced early-stage cement hydration, which may be promoted by the presence of fine suspended solids or increased ionic concentrations in reclaimed water [20].

In contrast, M-cSRW exhibited a flow of 185.18 mm and an initial setting time of 3.26 h. Although the setting time of M-cSRW was slightly longer than that of M-SRW, it remained shorter than that of M-DW. The higher flow and delayed setting behavior observed for M-cSRW relative to M-SRW can be attributed to the carbonation reaction, which converted dissolved calcium ions in the reclaimed water into calcium carbonate. This conversion partially reduced the concentration of calcium ions participating in early hydration reactions, thereby moderating the hydration process [21].

Overall, these results indicate that the use of reclaimed water significantly influences workability and setting behavior, whereas carbonation treatment tends to moderate these effects, resulting in more stable hydration behavior and setting times compared to untreated reclaimed water.

**Figure 9:**
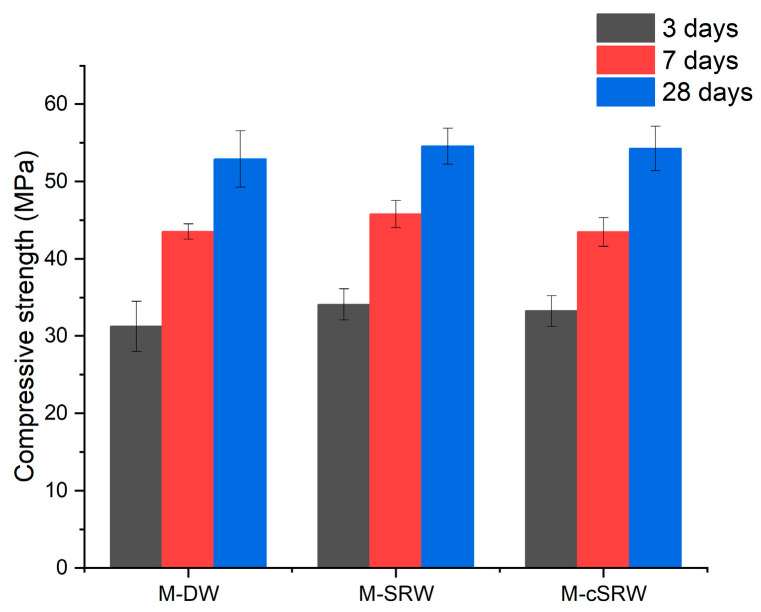
Compressive strength of mortars using reclaimed water as mixing water.

Figure 9 illustrates the compressive strength development of M-DW, M-SRW, and M-cSRW. The results show that both M-SRW and M-cSRW achieved equal or slightly higher strength compared to M-DW at all ages.

At 3 days, the compressive strength of M-DW was 31.25 MPa, whereas M-SRW and M-cSRW exhibited slightly higher strengths of 34.12 MPa and 33.25 MPa, respectively. At 7 days, M-SRW achieved the highest strength at 45.80 MPa, followed by M-DW (43.53 MPa) and M-cSRW (43.50 MPa), both of which showed comparable values. At 28 days, all specimens exhibited similar strength levels, with values of 52.92 MPa for M-DW, 54.58 MPa for M-SRW, and 54.28 MPa for M-cSRW, confirming that long-term strength was reliably maintained regardless of the mixing water condition.

These findings indicate that the use of SRW and cSRW does not adversely affect mechanical performance. The strength enhancement observed when reclaimed water was used can be attributed to accelerated hydration, promoted by fine particles present in the reclaimed water solids acting as nucleation sites for hydration products during the early stages of reaction [22]. This interpretation is consistent with the previously observed reduction in initial setting time.

### 3.4. Fresh Properties of Concrete Produced with Synthetic Reclaimed Water

**Figure 10:**
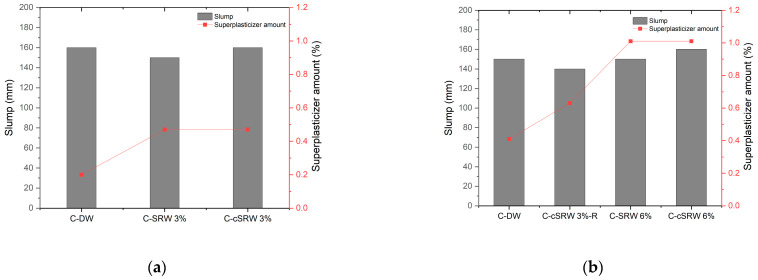
Slump and amount of superplasticizer in concrete with different mixing waters. (**a**) mixtures with 3% solid content (C-DW, C-SRW3%, and C-cSRW3%), (**b**) mixtures with increased solid content and cement substitution (C-cSRW3%-R, C-SRW6%, and C-cSRW6%).

Figure 10 compares the slump and superplasticizer (SP) dosage required to achieve a target slump of 160 mm in fresh concrete produced with SRW and cSRW. C-DW was used as the control mix.

In Figure 10a, the C-DW mix exhibited a slump of 160 mm with an SP dosage of 0.20%, whereas the C-SRW3% mix (with 3% solid content) showed a slightly reduced slump of 150 mm and required an SP dosage of 0.47%, indicating reduced workability. In contrast, the C-cSRW3% mix achieved a slump of 160 mm at the same SP dosage of 0.47%, which was identical to that of C-SRW3%, suggesting that carbonation of reclaimed water can enhance concrete workability.

Figure 10b presents the results for C-cSRW3%-R, in which 3% of the cement was substituted with solids contained in the reclaimed water. The control C-DW mix exhibited a slump of 150 mm at an SP dosage of 0.41%. In comparison, C-cSRW3%-R showed a slump below 140 mm at an SP dosage of 0.63%. The slumps of C-SRW6% and C-cSRW6% were 150 mm and 160 mm, respectively, at an SP dosage of 1.01%.

Overall, the use of reclaimed water instead of distilled water tended to reduce slump and increase the demand for superplasticizer. However, carbonation-treated reclaimed water generally improved flow characteristics compared to uncarbonated C-SRW at equivalent solid contents. These trends are consistent with those observed in the mortar tests and indicate that carbonation treatment of reclaimed water can effectively enhance the workability of fresh concrete. The observed improvement in workability can be attributed to the formation of these finely dispersed CaCO_2_ particles, whose filler and particle-packing effects have been widely reported to reduce water demand and enhance the fresh-state properties of cementitious systems [23]. In addition, the reduction in dissolved calcium ions following carbonation mitigated early flocculation of cement particles and moderated the initial hydration kinetics, which further contributed to higher flowability and delayed setting behavior. Therefore, the enhanced workability of the cSRW mixture is attributed to the combined effects of calcium ion stabilization and the physical contribution of finely dispersed CaCO_3_, rather than to pH reduction alone.

**Figure 11:**
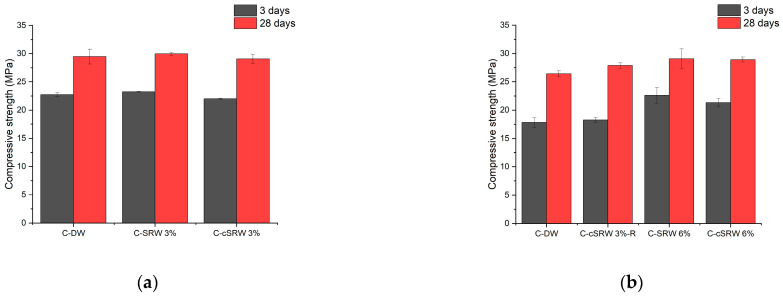
Compressive strength of concrete produced with carbonated reclaimed water at 3 and 28 days. (**a**) mixtures prepared with distilled water (C-DW), reclaimed water (C-SRW3%), and carbonated reclaimed water (C-cSRW3%) at a solid content of 3%, (**b**) mixtures incorporating higher solid contents and cement substitution, including C-SRW3%-R, C-SRW6%, and C-cSRW6%.

Figure 11a,b present the compressive strength results at 3 and 28 days. In Figure 11a, the 3-day and 28-day compressive strengths of C-DW were 22.73 MPa and 29.47 MPa, respectively. The C-SRW3% mix showed slightly improved strength values of 23.23 MPa at 3 days and 29.97 MPa at 28 days, while the C-cSRW3% mix yielded strengths of 22.00 MPa and 29.07 MPa, respectively, which are comparable to those of the C-DW control. Since the differences in compressive strength fall within the margin of experimental error, it can be concluded that the use of reclaimed water, regardless of carbonation treatment, results in comparable or slightly enhanced compressive strength relative to distilled water.

Figure 11b presents the results for C-cSRW3%-R. Despite a 3% reduction in cement content, C-cSRW3%-R achieved compressive strengths of 18.28 MPa at 3 days and 27.86 MPa at 28 days, both higher than those of C-DW (17.83 MPa and 26.40 MPa, respectively). The C-SRW6% and C-cSRW6% mixes exhibited compressive strengths of 22.62 MPa and 21.32 MPa at 3 days, and 29.08 MPa and 28.92 MPa at 28 days, respectively, indicating that higher solid contents did not adversely affect strength development. The comparable or even higher strength observed in the cSRW3%-R mixture, despite partial replacement of cement by the solids present in reclaimed water, can be attributed to several combined mechanisms. First, the solids in reclaimed water mainly consist of finely dispersed cementitious residues and calcium-rich particles, which act as micro-fillers. These fine particles improve particle packing density and reduce capillary porosity, leading to a denser microstructure even with a slightly reduced cement content. Second, as re-ported in previous studies [22], fine particles in the submicron to micron range can serve as heterogeneous nucleation sites for hydration products, accelerating early hydration and enhancing the formation of C–S–H. This nucleation effect compensates for the reduced cement content, particularly at early ages. In addition, carbonation treatment stabilizes dissolved calcium species by converting them into finely dispersed CaCO_3_, which further contributes to the filler effect and improves the efficiency of hydration product precipitation.

Overall, these results confirm that the use of reclaimed water does not compromise the compressive strength of concrete. Carbonation treatment and increased solid content also did not negatively influence strength development. In particular, C-cSRW3%-R, in which a portion of cement was replaced by solids present in reclaimed water, exhibited comparable or even superior strength, demonstrating the potential of carbonated reclaimed water for use in sustainable concrete production.

### 3.5. Discussion and Practical Implications

In this study, the carbonation degree of RW and its potential use as mixing water in concrete were assessed under controlled laboratory conditions through a simplified CO_2_ injection method.

It should be noted that the experimental conditions used in this study are not fully compatible with the scale and constraints of actual concrete production processes. First, the CO_2_ injection rate of 3 L/min is excessively high relative to the reclaimed water volume of 1 L. To achieve a comparable level of CO_2_ sequestration performance in real concrete applications, technical strategies are required to enhance reaction efficiency—such as controlling CO_2_ solubility and injection rate, adjusting reaction time, and optimizing mixing conditions. Second, although this study evaluated the carbonation degree using SRW composed of controlled proportions of cement solids and fine aggregate, actual RW represents a more complex aqueous system containing various impurities, such as residual admixtures, ultrafine particles, dissolved salts, and organic matter. These components may influence CO_2_ reactivity, the precipitation behavior of calcium carbonate, and the stability of hydration products.

Therefore, further investigations are necessary to examine the reaction characteristics associated with variations in actual RW composition and to develop appropriate strategies for reaction control. In addition, a comprehensive life-cycle assessment that quantify both the energy consumption and the net CO_2_ balance associated with CO_2_ injection into reclaimed water under realistic operational conditions needs to be conducted.

While this study focused on quantifying CaCO_3_ formation through TG analysis and evaluating the mechanical properties of mortar and concrete, practical implementation requires further investigations into the long-term stability of the precipitated CaCO_3_, its impact on steel corrosion, and the overall durability of concrete produced using cSRW. In particular, future work should examine the use of catalysts, reactor design optimization, and multi-stage CO_2_ injection strategies to enhance CO_2_ uptake and process efficiency.

In conclusion, to enhance the practical applicability of mineral carbonation technology for RW, laboratory-scale reactivity assessments should be complemented by pilot-scale field trials, economic feasibility analyses, and comprehensive life-cycle assessments (LCA) to validate both the technical viability and carbon-reduction potential of the system.

## 4. Conclusions

This study investigated the feasibility of simultaneously sequestering CO_2_ in reclaimed water and reusing the treated water as mixing water for mortar and concrete.

- Experimental results using CO_2_-enriched nanobubble water and gaseous CO_2_ demonstrated that cementitious solids present in reclaimed water effectively react with CO_2_ to form stable calcium carbonate. Using a simple CO_2_ injection method, up to 10.5 g of CO_2_ per kilogram of reclaimed solids was fixed.
- The use of reclaimed water as mixing water slightly reduced flowability and increased the demand for superplasticizer compared with distilled water. However, carbonation treatment of reclaimed water improved workability and mitigated rapid initial setting behavior.
- Compressive strength development of mixtures prepared with reclaimed water was comparable to, or in some cases greater than, that of the control mixture. In particular, the C-cSRW3%-R mixture, in which 3% of the cement was partially replaced with solids from carbonated reclaimed water, maintained stable mechanical performance.
- These results confirm that CaCO_3_ formed through the carbonation of reclaimed water can be stably retained within the concrete matrix, indicating that carbonation-treated reclaimed water can function as a viable medium for CO_2_ storage and offer additional processing benefits.

Overall, this approach presents a promising carbon-neutral strategy for the cement and concrete industry by enabling direct CO_2_ sequestration within construction materials. Further studies are required to validate field applicability and long-term durability.

## Figures and Tables

**Table 1:** Compositions of ordinary Portland cement.

Chemical Composition		Mineralogical Composition	
CaO	62.8	C_3_S (3CaO·SiO_2_)	54.7
SiO_2_	20.3	C_2_S (2CaO·SiO_2_)	15.3
SO_3_	2.2	C_4_AF (3CaO·Al_2_O_3_)	12.4
Al_2_O_3_	4.7	C_3_A (4CaO·Al_2_O_3_·Fe_2_O_3_)	5.9
MgO	2.5	Calcite (CaCO_3_)	4.2
Fe_2_O_3_	3.3	Gypsum (CaSO_4_·2H_2_O)	2.2
Na_2_O	0.13	Bassanite (CaSO_4_·0.5H_2_O)	2.3
K_2_O	0.74	Quartz (SiO_2_)	1.6
TiO_2_	0.31	Arcanite (K_2_SO_4_)	1.4
Others	3.17		
LOI	2.79		
sum	100	sum	100

LOI: Loss on Ignition

**Table 2:** Objects of investigation and corresponding sample labels used in this study.

Type	Short Names	Description
Water	CBW	CO_2_-enriched nanobubble water
	RW	Actual reclaimed water
	SRW	Synthetic reclaimed water
Mortar	M-DW	Distilled water (control)
	M-SRW	Synthetic reclaimed water (3 wt.% of binder)
	M-cSRW	Carbonated synthetic reclaimed water (3 wt.% of binder)
Concrete	C-DW	Distilled (control)
	C-SRW3%	Synthetic reclaimed water (3 wt.% of binder)
	C-cSRW3%	Carbonated synthetic reclaimed water (3 wt.% of binder)
	C-cSRW3%-R	Carbonated synthetic reclaimed water (3 wt.% of binder) and 3% cement replacement
	C-SRW6%	Synthetic reclaimed water (6 wt.% of binder)
	C-cSRW6%	Carbonated synthetic reclaimed water (6 wt.% of binder)

**Table 3:** Mix proportions of mortar using reclaimed water as mixing water (by mass).

	Type	*w*/*c*	Distilled Water	RW		Cement	Fine Aggregate	Coarse Aggregate
				Water	Solid Content			
Mortar	M-DW	0.5	0.5	0	0	1.0	3.0	-
	M-SRW		0	0.50	0.03			
	M-cSRW		0	0.50	0.03			

**Table 4:** Mix proportions of concrete using reclaimed water as mixing water (kg/m^3^).

	Type	*w*/*c*	Distilled Water (kg)	RW		Cement (kg)	Fine Aggregate (kg)	Coarse Aggregate (kg)
				Water (kg)	Solid Content (kg)			
Concrete	C-DW	0.5	185	0	-	370	845	875
	C-SRW3%		0	185	11.1	370		
	C-cSRW3%		0	185	11.1	370		
	C-cSRW3%-R		0	185	11.1	358.9		
	C-SRW6%		0	185	22.2	370		
	C-cSRW6%		0	185	22.2	370		

**Table 5:** TG analysis of cement powder reacted with CO_2_ nano-bubble water.

Mixing Water	Solid Content (%)	Residue at 500 °C (%) ^a^	Residue at 850 °C (%) ^b^	CO_2_ Uptake (^a-b^) (%)	Net CO_2_ Uptake (%) **	pH	Reacted CO_2_/ CBW (mg/L)
DW * (Control)	5	97.65	95.66	1.99	-	11.95	-
CBW	1	97.04	85.54	11.51	9.52	10.75	952
	3	96.70	90.80	5.90	3.92	11.57	1173
	5	97.11	92.79	4.32	2.33	11.23	1165
	7	97.72	94.48	3.25	1.26	11.55	882
	10	97.20	94.03	3.17	1.19	11.67	1180
	15	97.75	95.29	2.46	0.47	11.82	705

* DW: distilled water, CBW: CO_2_ nano-bubble water. ** Net CO_2_ uptake exclude the CO_2_ content of the control (DW)

**Table 6:** CO_2_ uptake of RW-A as a function of CO_2_ injection time.

CO_2_ Injection Time (h)	Solid Content (%)	Residue at 500 °C (%) ^a^	Residue at 850 °C (%) ^b^	CO_2_ Uptake (^a-b^) (%)	Net CO_2_ Uptake (%)
control	5	87.80	84.38	3.42	-
0.5		92.31	70.19	22.12	18.70
1		92.34	69.76	22.58	19.16
3		92.49	69.57	22.92	19.50

**Table 7:** TG results before and after carbonation of actual reclaimed water samples (5 wt.% solid content; all percentages are expressed as weight percentages, wt.%).

Sample	Residue at 500 °C (%) ^a^	Residue at 850 °C (%) ^b^	CO_2_ Uptake (^a-b^) (%)	Net CO_2_ Uptake (%)	Net CO_2_ Uptake Per 1 L of RW (mg/L)
RW A	87.80	84.38	3.42	-	-
RW A CO_2_	92.22	69.68	22.55	19.13	9560
RW B	86.12	81.89	4.23	-	-
RW B CO_2_	91.40	69.40	22.00	17.77	8890
RW C	85.77	80.15	5.62	-	-
RW C CO_2_	93.78	73.98	19.80	14.18	6990

**Table 8:** TG analysis results before and after CO_2_ carbonation of synthetic reclaimed water samples (5 wt.% solid content; all percentages are expressed as weight percentages, wt.%).

Sample (C:S)	Residue at 500 °C (%)	Residue at 850 °C (%)	CO_2_ Uptake (%)	Net CO_2_ Uptake (%)	Net CO_2_ Uptake Per 1 L of RW (mg/L)	Net CO_2_ Uptake Per Cement Content in 1 L of RW (%)
10-0	92.36	87.92	4.44	-	-	-
10-0 CO_2_	94.99	69.27	25.72	21.28	10,530	21.06
9-1	92.99	88.82	4.17	-	-	-
9-1 CO_2_	95.53	71.87	23.67	19.50	9640	21.42
8-2	94.19	90.37	3.82	-	-	-
8-2 CO_2_	95.85	75.26	20.59	16.77	8305	20.76
7-3	94.46	90.99	3.47	-	-	-
7-3 CO_2_	96.26	79.19	17.07	13.59	6760	19.31
6-4	94.77	91.66	3.10	-	-	-
6-4 CO_2_	96.78	82.87	13.90	10.80	5380	17.93

**Table 9:** Flow and initial setting time of mortar sample.

Type	Mortar Flow (mm)	Initial Setting Time (hr)
DW	208.57	5.66
SRW	180.71	3.09
cSRW	185.18	3.26

## Data Availability

The original contributions presented in this study are included in the article. Further inquiries can be directed to the corresponding author.
